# *Bt*-maize (MON810) and Non-GM Soybean Meal in Diets for Atlantic Salmon (*Salmo salar* L.) Juveniles – Impact on Survival, Growth Performance, Development, Digestive Function, and Transcriptional Expression of Intestinal Immune and Stress Responses

**DOI:** 10.1371/journal.pone.0099932

**Published:** 2014-06-12

**Authors:** Jinni Gu, Anne Marie Bakke, Elin C. Valen, Ingrid Lein, Åshild Krogdahl

**Affiliations:** 1 Department of Basic Sciences and Aquatic Medicine, NMBU School of Veterinary Medicine, Norwegian University of Life Sciences (NMBU), Oslo, Norway; 2 Nofima AS, Sunndalsøra, Norway; Catalan Institute for Water Research (ICRA), Spain

## Abstract

Responses in Atlantic salmon (*Salmo salar* L.) juveniles (fry) fed diets containing genetically modified maize (*Bt*-maize, MON810) expressing Cry1Ab protein from first-feeding were investigated during a 99-day feeding trial. Four experimental diets were made; each diet contained ∼20% maize, either *Bt*-maize or its near-isogenic maternal line (non-GM maize). One pair was fishmeal-based while the other pair included standard (extracted) soybean meal (SBM; 16.7% inclusion level), with the intention of investigating responses to the maize varieties in healthy fish as well as in immunologically challenged fish with SBM-induced distal intestinal inflammation, respectively. Three replicate tanks of fry (0.17±0.01 g; initial mean weight ± SEM) were fed one of the four diets and samples were taken on days 15, 36, 48 and 99. Survival, growth performance, whole body composition, digestive function, morphology of intestine, liver and skeleton, and mRNA expression of some immune and stress response parameters in the distal intestine were evaluated. After 99 days of feeding, survival was enhanced and the intended SBM-induced inflammatory response in the distal intestine of the two groups of SBM-fed fish was absent, indicating that the juvenile salmon were tolerant to SBM. Mortality, growth performance and body composition were similar in fish fed the two maize varieties. The *Bt*-maize fed fish, however, displayed minor but significantly decreased digestive enzyme activities of leucine aminopeptidase and maltase, as well as decreased concentration of gut bile salts, but significantly increased amylase activity at some sampling points. Histomorphological, radiographic and mRNA expression evaluations did not reveal any biologically relevant effects of *Bt*-maize in the gastrointestinal tract, liver or skeleton. The results suggest that the Cry1Ab protein or other compositional differences in GM *Bt*-maize may cause minor alterations in intestinal responses in juvenile salmon, but without affecting overall survival, growth performance, development or health.

## Introduction

Since the first commercialization in 1996, safety issues regarding the introduction of GM crops into food and feed have been debated. Among the globally available GM crops, *Bt*-maize (MON810) is the only GM maize event authorized and cultivated in the EU and is mainly used in animal feeds [1]. MON810 expresses Cry1Ab protein, which confers maize resistance to the European corn borer, *Ostrinia nubilalis*. The Cry proteins are a group of endotoxins produced by different strains of soil bacterium *Bacillus thuringiensis* (*Bt*) and have putative species-specific toxicity for insects [2]–[3]. The Cry toxins are considered non-toxic to higher animals [4]–[5] and the *Bt*-crops have been suggested to be as safe and nutritious as their conventional/commercial counterparts for various animals in some [6]–[14], but not all studies. Among the latter, Cry1A toxins have been reported to bind to the mammalian intestine's mucosal surfaces [5], [15], and suggested to thereby elicit humoral and mucosal immune responses in mice [16]–[20]. Along with reported biological responses to *Bt*-maize, concerns regarding possible allergenicity of GM plant crops, in particular the transgenic proteins, have been raised [21]–[22]. Transgenic proteins undergo different post-translational modifications following integration of the transgenic DNA into a foreign organism's genome [23], which may alter their allergenic potential. The authors of these studies also suggest that GM crops may potentiate pre-existing allergies.

In concert, all these various concerns have elicited uncertainty regarding long term ramifications of GM crop consumption to the health and longevity of animals and humans. In addition to the extensive risk assessment prior to market authorization, post-market monitoring (PMM) may now be required by the EU commission, based on the specific results of the pre-market risk assessment, to address these concerns. When required, PMM is considered necessary to confirm the safety of the products in the long term, and to increase the probability of detecting unintended effects [24]. Implementation of PMM is the responsibility of the company seeking market release of their GM products (authorization holder). Finding suitable biomarkers for GM exposure are potentially useful for PMM. A 7^th^ Framework Programme EU-project entitled “Biomarkers for post market monitoring of short and long-term effects of genetically modified organisms on animal and human health” (GMSAFOOD) was funded to investigate whether such biomarkers could be identified. The project used *Bt*-maize as the authorized GM model. Model animals included mice, rats, pigs and fish at various stages of development.

Inclusion of plant crops in commercial diets for farmed fish is increasing in an effort to reduce dependence on limited marine resources such as fishmeal and fish oil, and to improve cost efficiency and sustainability of the aquaculture industry. This includes piscivorous species such as Atlantic salmon (*Salmo salar* L.). A large share of the global market of some plant crops, such as maize and soybeans, are now genetically modified (GM). To assess its safety, feeding trials with GM *Bt*-maize in post-smolt [13], [25]–[28] and juvenile [10]–[11], [29] Atlantic salmon diets, as well as in diets for a model fish species, zebrafish (*Danio rerio*) [12], [14], have been conducted. Although no clearly negative health effects were observed in these studies, the salmon fed *Bt*-maize-containing diets displayed some differences in performance parameters and functional responses compared to control non-GM maize diets, including reduced feed intake and growth despite elevated activity of maltase in mid and distal intestine [25], and elevated gene expression of stress-related heat shock protein (HSP) 70 and superoxide dismutase (SOD) in the liver [26]. More recently and within the GMSAFOOD project, post-smolt (seawater stage) Atlantic salmon fed a 20% inclusion level of *Bt*-maize for 90 days displayed reductions in digestibility of protein and mineral, retention efficiency of lipid and energy, as well as activity of leucine aminopeptidase (LAP) in proximal intestine. Gene expression of the T cell marker CD4 and the cytokine interferon-γ in the distal intestine was increased [28]. In an effort to explore whether a pre-existing hypersensitivity response would alter responses to *Bt*-maize, experimental groups were included that were simultaneously exposed to extracted soybean meal (SBM) in their diets, which causes an apparently T-cell-mediated type IV hypersensitivity response in the distal intestine of salmon [30]-[36]. The results suggested that *Bt*-maize potentiated oxidative cellular stress in the inflamed intestine, as indicated by increased transcription level of superoxide dismutase (SOD) and heat shock protein 70 (HSP70) [28]. However, no specific biomarkers for GM exposure or responses to exposure to use for PMM were identified.

The present paper documents the results from a 99-day feeding trial on developing Atlantic salmon juveniles fed *Bt*-maize-containing diets from first-feeding. Mirroring the design of the prior study in post-smolts [28], a two-by-two factorial design was implemented, in which near-isogenic maternal line of non-GM maize or *Bt*-maize were added to both a diet containing fishmeal as the sole protein source and a SBM-containing diet expected to induce inflammation in the distal intestine. Survival, growth performance, activities of intestinal and pancreatic enzymes, bile salt content, morphological examination of the gastrointestinal tract, liver and skeleton, as well as gene expression of various immune cell markers, cytokines, and stress response parameters in the distal intestine were evaluated at various time points during the course of the feeding trial. The aims were to investigate 1) unintended *Bt*-maize responses in general, 2) whether challenged fish with an intestinal inflammation would alter the response to dietary *Bt*-maize, as well as 3) whether any response parameters could be suitable biomarkers for GM responses to exposure. However, the data indicated that the juvenile salmon did not respond with intestinal inflammation to SBM as more developed salmon do, suggesting tolerance to SBM. Thus the second aim was not achieved.

## Materials and Methods

### Ethics statement

Rearing of the fish was conducted at Nofima's Research Station (Sunndalsøra, Norway), which is an approved research facility by Norwegian Animal Research Authority (NARA) and operates in accordance with the Norwegian Regulations of 17 June 2008 No. 822: Regulations relating to Operation of Aquaculture Establishments (Aquaculture Operation Regulations). Up to sacrifice and sampling, the fish were treated as production fish in accordance with aforementioned Aquaculture Operation Regulations. Prior to sampling, the fish were humanely anaesthetized before euthanasia in accordance with the Norwegian Animal Welfare act. Hence, no NARA approval was required according to Dr. G Baeverfjord (Nofima), appointed by NARA.

### Feed ingredients, experimental diets and feeding

Two maize types, *Bt*-maize (MON810) and its near-isogenic maternal line (non-GM maize) derived from planted seed varieties PR34N44 and PR34N43, respectively, were provided by Pioneer (Johnston, IA, USA). They were grown simultaneously in neighboring fields in Spain. Details regarding nutrient composition, pesticide and mycotoxin levels, and level of *Cry1Ab* gene insert in the two maize meals have been previously reported [37]-[38]. All the maize grains were dried and ground just prior to diet preparation to obtain whole maize meal. Feeds were prepared by Nofima AS in Bergen, Norway. Diet formulations are given in Table 1. Extracted soybean meal was obtained from Denofa AS (Fredrikstad, Norway) and was certified to be non-GM (www.denofa.com). The diets were balanced regarding vitamins and minerals according to estimated requirements [39] and were optimized to give diets with equal protein:energy ratios of 25 g/MJ. As a result of the higher fibre content of SBM compared to fishmeal, the SBM-containing diets were somewhat lower in protein and less energy dense. The lower energy density was expected to increase feed consumption. Therefore a somewhat lower level of maize was added to the SBM-containing diets, which was expected to result in more equal exposure to maize across the experimental diets. The feed was extruded, crumbled and sieved into three particle sizes: 0.6 mm, 0.9 mm and 1.3 mm.

**Table 1:** Formulation and proximate composition (as fed basis) of the experimental diets.

	Non-GM maize	*Bt*-maize	Non-GM maize+SBM	*Bt*-maize+SBM
*Ingredients (g kg^−1^)*				
Fish meal (58/09)^a^	706	706	564	564
Non-GM maize	200	-	167	-
*Bt*- maize (MON810)	-	200	-	167
Extracted SBM (239/08)^b^	-	-	167	167
NorSalmOil^c^	70	70	80	80
Vitamin mix^d^	20	20	19	19
Mineral mix^e^	4	4	4	4
Carophyll Pink 10%	0.2	0.2	0.2	0.2
Protein (g)/engery (MJ)	24.6	24.7	25.0	25.0
*Proximate composition (g kg^−1^)*				
Dry matter	947	932	942	931
Crude protein	519	501	485	479
Crude lipid	164	155	154	146
Gross energy (MJ kg^−1^)^f^	21.6	21.1	21.3	20.8
Protein (g)/energy (MJ)	24.0	23.7	22.8	23.0

a Norseco-LT, Norsildmel, Bergen, Norway.

b Extracted soybean meal, Denofa As, Fredrikstad, Norway.

c NorSalmOil, Norsildmel, Bergen, Norway.

d Normin AS, Hønefoss, Norway. Diets supplied with following vitamins per kg diet: vitamin D3, 3000 I.E; vitamin E (Rovimix, 50%), 160 mg; thiamine, 20 mg; riboflavin, 30 mg; pyridoxine-HCl, 25 mg; vitamin C (Riboflavin Stay C 35%), 200 mg; calcium pantothenate, 60 mg; biotin, 1 mg; folic acid, 10 mg; niacin, 200 mg; vitamin B_12_, 0.05 mg; menadione bisulphate, 20 mg.

e Normin AS, Hønefoss, Norway. Diets supplied with following minerals per kg diet: magnesium, 750 mg; potassium, 800 mg; zinc, 120 mg; iron, 60 mg; manganese, 30 mg; copper, 6 mg and selenium; 0.3 mg.

f Gross energy was calculated using the energy concentrations of 39.5 for lipid, 23.6 for protein, and 17.2 kJ/g for carbohydrates (carbohydrate levels in diets were calculated as: 100 – [water + crude protein + crude lipid + ash]).

### Experimental design and facilities

The experiment was carried out using a 2×2 factorial design with four diet groups. The factors GM and SBM inclusion were tested separately and in combination. The fry of SalmoBreed origin with an approximate initial weight of 0.17±0.01 g (mean ± standard error of the mean) were randomly allocated, ca. 1200 fry per tank, to 12 tanks, 60 cm in diameter. Each of the four experimental diets was fed to triplicate tanks of fish. The fresh water level was kept at 19 cm at initiation of the feeding period and later elevated to 32 cm when the fish started to swim upwards in the water column. The feed pellet size at start was 0.6 mm, and was subsequently adjusted to 0.9 mm and 1.3 mm as the fish grew to 1 and 3 g, respectively. The tanks were supplied with filtered fresh water at approximately 12°C. The fish were fed by automatic belt feeders set to supply feed every 10 min, and the feeding level was 20% in excess of estimated feed requirement. Due to the small size of the feed pellets, feed intake could not be accurately measured. Light was provided continuously. All mortalities were recorded.

### Sampling procedure

Samplings were conducted following 15, 36, 48, and 99 days of exposure to the experimental diets. Sampled fish were in the fed state as fasting may affect physiological parameters [40] and diet-induced inflammatory changes [30] in the intestine. Randomly selected fish were anaesthetized and sacrificed by a lethal dose of tricaine methane-sulfonate (MS222; Argent Chemical Laboratories, Inc., Redmond, WA, USA) prior to examination and dissection. Body weight and fork length were measured for 10 fish per tank. Depending on the size of fish, 2–20 whole fish and/or dissected intestinal sections were pooled for assays of digestive enzyme, mRNA expression and whole body chemical analysis. When the fish were large enough to be dissected, i.e. at 48 days of dietary exposure, different regions of the intestinal tract – proximal (PI), mid (MI) and distal (DI) intestine – were collected as described earlier [41]. Samples for histological examination were fixed in 4% buffered formaldehyde solution for 24h and subsequently stored in 70% ethanol until further processing. For mRNA expression investigations, the dissected DI were kept in RNAlater for 24h and subsequently stored at −20°C. Samples for whole body composition and digestive enzyme analyses were frozen in liquid nitrogen and stored at −80°C. Fish sampled for skeletal development examination were single-frozen on a flat board and stored at −80°C until radiography.

### Chemical analyses

Diets were analyzed for dry matter, crude protein, and crude lipid. At the end of the 99 day feeding trial, one pooled sample of 20 whole fish per tank was analyzed for whole body composition of dry matter, crude protein, crude lipid, and ash. Analyses were performed at Nofima AS (Sunndalsøra, Norway) following standard methods. In short, dry matter was measured by drying at 105°C for 16–18 h. Nitrogen (N) was analyzed according to semi-micro-Kjeldahl method with Kjeltec-Auto System (Tecator, Höganäs, Sweden) and crude protein was calculated as N*6.25. Crude lipid was determined in a Fosstec analyser (Tecator, Höganäs, Sweden) after diethyl ether extraction. For ash, samples were weighed before and after burning at 550°C.

### Digestive enzyme activities and bile acid concentrations

One pooled sample of 15 whole fish per tank on days 15 and 36, and 10 whole fish per tank on days 48 and 99, respectively, were analysed for activities of pancreatic enzymes trypsin and amylase, brush border membrane enzymes leucine aminopeptidase (LAP) and maltase, as well as bile acid concentration. At the last two samplings, one pooled sample of 10 dissected intestinal sections (with intestinal content) per tank was also analysed for the above-mentioned parameters. On the day 48 sampling, the small size of the intestines made distinguishing the MI from the DI intestinal regions difficult. Thus the MI and DI (MI+DI) were analysed together, while the PI, with its visible pyloric caeca, was analysed separately. On day 99, the DI was analysed separately, while MI and PI samples (PI+MI) were analysed together.

LAP and maltase activities, investigated to assess intestinal function, were analyzed following the method described previously [40]. The frozen samples were thawed and homogenized (1∶20 w/v) in ice-cold 2 mM Tris/50 mM mannitol, pH 7.1, containing serine protease inhibitor Pefabloc SC (No. 11179, Pentapharm Ltd). The LAP activity was measured colorimetrically using L-leucine β-naphthylamide hydrochloride (No. L0376, Sigma-Aldrich) as the substrate. LAP activity is expressed as mmol substrate hydrolyzed as related to unit time (h) and kg body weight. Maltase activity was analyzed using maltose as substrate. Maltase activity is expressed as mmol substrate hydrolysed as related to unit time (min) and kg body weight.

For trypsin activity analyses, the frozen tissue with intestinal content was homogenized and suspended in dH_2_O. Trypsin activity was measured colorimetrically according to Kakade et al. [42], using the substrate benzoyl-arginine-p-nitroanilide (BAPNA; No. B-4875, Sigma-Aldrich). Activity is expressed as change in optic density (OD) related to mg tissue homogenate. Amylase activity was measured using a Randox amylase assay kit (AY892, Randox Laboratories Ltd., Crumlin, UK) by hydrolysis of benzylidene-blocked p-nitrophenyl maltoheptaoside (pNPG7). The amylase activity is expressed in mU g^−1^ tissue homogenate.

The bile salt concentration in samples was determined using the Enzabile kit from Nycomed Pharma AS Diagnostics (Oslo, Norway) and expressed as mg g^−1^ tissue homogenate.

### Histology

Formalin-fixed liver, PI and DI samples from four fish per tank, i.e. 12 fish per diet, sampled on day 99 were routinely dehydrated in ethanol, equilibrated in xylene and embedded in paraffin according to standard histological procedures. Sections of 3–5 µm were stained with haematoxylin and eosin (H&E) and blindly evaluated under a light microscope (Carl Zeiss, Inc. UK) by a trained veterinarian. Morphology of PI and DI was evaluated according to the criteria previously described in Atlantic salmon [30], [43]: (1) length of mucosal folds; (2) the degree of vacuolization in the absorptive cells (enterocytes); (3) width and cellularity of lamina propria and submucosa; (4) frequency of goblet cells. The degree of histological changes was graded as normal, mild, moderate or severe.

### Skeletal development examination

Radiography of frozen fish from the day 99 sampling, 50 to 58 individuals per tank, was conducted in a semi-digital system, with a Giotto Image (IMS, Bologna, Italy) mammography X-ray source combined with an FCR Profect image plate reader and FCR Console (Fuji Medical Inc., Japan). The system comprises automated image enhancement-procedures for contrast enhancement and edge visualization. The skeletal morphology was evaluated by trained personnel according to the following criteria: (1) fusions and fusion-associated changes; (2) compressed vertebrae in neck; (3) complex deformities, consisting of a mixture of fusions, compressions and distorted shapes; (4) head deformities.

### Quantitative real-time PCR (qPCR)

Real-time quantitative PCR analyses were limited to the DI, where possible interactions in SBM-fed (immune-sensitised) salmon would be expected. Transcriptional expression of T helper cell marker CD4, the cytokines interleukin 17a (IL17a) and interferon gamma (IFNγ), proliferating cell nuclear antigen (PCNA), and heat shock protein (HSP) 70 in the DI tissue were assessed. Total RNA was extracted from dissected DI on the day 99 sampling from three fish per tank, i.e. nine animals per diet. RNA purification and quality control, DNase treatment, complementary DNA synthesis, primer optimisation and quantitative PCR assays were performed as described in detail elsewhere [44]. Quantitative PCR primers were obtained from the literature or designed using Primer3 (http://frodo.wi.mit.edu/primer3). See Table 2 for details. Elongation factor 1α (EF1A), β-actin (ACTB), RNA polymerase II (RNAPOLII), hypoxanthine phosphoribosyltransferase 1 (HPRT1), ribosomal protein S20 (RPS20) and glyceraldehyde-3-phosphate dehydrogenase (GAPDH) were evaluated for use as reference genes by ranking expression levels according to their stability, as described previously [44]. The geometric mean of three stably expressed genes (RPS20, ACTB and HPRT1) was used as the normalisation factor. Mean normalised expression of the target genes was calculated from raw Cq values [45].

**Table 2:** Primer pair sequences, amplicon size (AS in basepairs [bp]), annealing temperature (AT), efficiency (E) and Genbank accession number (Gen. Acc. No.) for genes used for quantitative real-time PCR.

Gene	5′- 3′ primer sequence		AS (bp)	AT (°C)	E	Gen. Acc. No.	References
	Forward	Reverse					
CD4	GAGTACACCTGCGCTGTGGAAT	GGTTGACCTCCTGACCTACAAAGG	123	60	1.85	DQ867018	36
IL17a	TGGTTGTGTGCTGTGTGTCTATGC	TTTCCCTCTGATTCCTCTGTGGG	136	60	2.00	GW574233	36
IFNγ	CTAAAGAAGGACAACCGCAG	CACCGTTAGAGGGAGAAATG	159	60	1.93	FJ263446	36
PCNA	TGAGCTCGTCGGGTATCTCT	GTCCTCATTCCCAGCACACT	170	55	2.00	BT056931	28
HSP70	CCCCTGTCCCTGGGTATTG	CACCAGGCTGGTTGTCTGAGT	121	60	1.97	BG933934	28
ACTB	CAAAGCCAACAGGGAGAAGATGA	ACCGGAGTCCATGACGATAC	133	60	1.90	AF012125	44
EF1A	GTGCTGTGCTTATCGTTGCT	GGCTCTGTGGAGTCCATCTT	148	60	1.91	AF321836	44
GAPDH	AAGTGAAGCAGGAGGGTGGAA	CAGCCTCACCCCATTTGATG	96	60	1.85	BT050045	44
HPRT1	CCGCCTCAAGAGCTACTGTAAT	GTCTGGAACCTCAAACCCTATG	255	60	1.99	BT043501	44
RNAPOLII	CCAATACATGACCAAATATGAAAGG	ATGATGATGGGGATCTTCCTGC	157	60	1.80	BG936649	44
RPS20	AGCCGCAACGTCAAGTCT	GTCTTGGTGGGCATACGG	98	60	1.97	AY953432	44

CD4, cluster of differentiation 4; IL17a, interleukin 17a; IFNγ, interferon gamma; PCNA, proliferating cell nuclear antigen; HSP70, heat shock protein 70; ACTB, β-actin; EF1A, elongation factor 1α; GAPDH, glyceraldehyde-3-phosphate dehydrogenase; HPRT1, hypoxanthine phosphoribosyltransferase 1; RNAPOLII, RNA polymerase II; RPS20, ribosomal protein S20.

### Calculations

Condition factor K (bw  =  body weight, fl  =  fork length): *K* = bw/fl^3^

Specific growth rate SGR (Wf  =  final weight, Wi  =  initial weight, t  =  time in days): 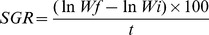



### Statistics

The data were statistically evaluated using JMP version 9.0 (2010) Statistical Discovery™ (SAS Institute Inc.). Most of the results were subjected to two-way ANOVA with GM and SBM inclusion as the class variables and performed on calculated tank means or results from pooled samples (n = 3 tanks for each dietary treatment) based on a variable number of sampled individuals per tank depending on sampling time and parameter (see respective sections above for details). However, the histological scores were compared using non-parametric contingency analysis and performed on data from 12 fish per dietary treatment (n = 12). All reported *p*-values are two-sided and significance was set at *p*<0.05. Trends/tendencies towards significance are discussed when *p*-values were in the range of 0.05–0.10.

## Results

Analyzed proximate compositions of the experimental diets were similar to expected compositions (Table 1). The protein:energy ratios of the four diets were close to the predicted 25 g/MJ. SBM-containing diets were slightly lower in crude protein and lipid levels, and somewhat higher in carbohydrate, also as expected (see Materials and methods section Feed ingredients, experimental diets and feeding).

Very low mortality was observed among the fish throughout the experiment with a cumulative mortality of ≤2.5% (pooled standard error 0.33), indicating high quality feed and husbandry conditions. Interestingly and contrary to studies in more mature salmon, survival and performance of the juveniles was enhanced by addition of SBM to the experimental diets and no SBM-induced inflammatory changes in the distal intestine were observed. As this was an unexpected finding with implications for the interpretation of the results regarding effects of the *Bt*-maize, SBM effects are briefly described first before the focus is shifted to GM effects and interactions between GM and SBM.

### Effects of SBM

**Figure 1:**
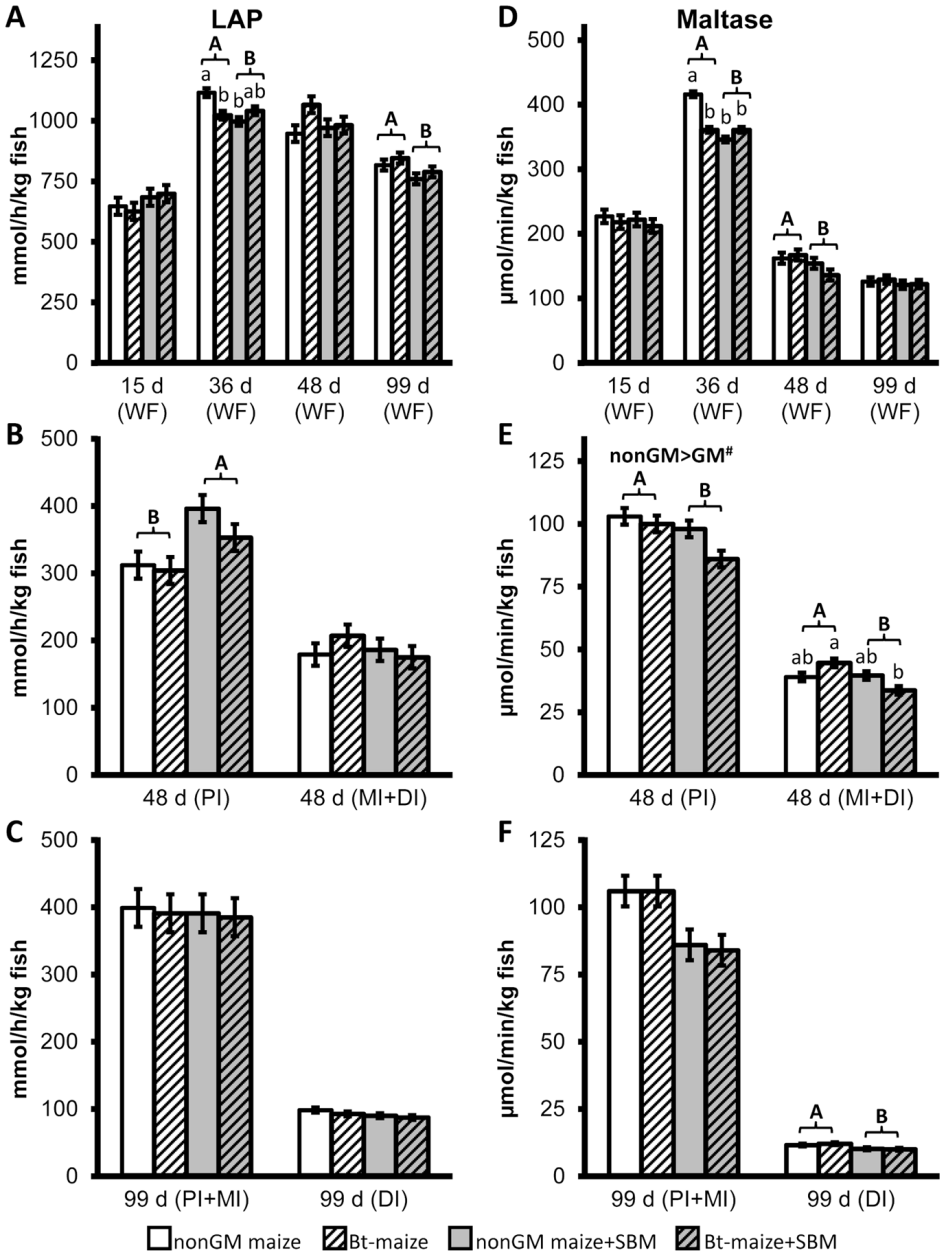
Mean activities of leucine aminopeptidase (LAP) in mmol substrate hydrolysed per hour per kg body weight (A, B and C) and maltase in µmol substrate hydrolysed per min per kg body weight (D, E and F) in whole fish and intestinal sections of Atlantic salmon juveniles fed non-GM maize or GM maize (*Bt*-maize) without or with soybean meal (SBM) from the first-feeding to day 99. Means ± pooled standard errors were calculated from pooled samples of 10–15 fish per tank, three replicate tanks per treatment group (n = 3; for more details see Materials and methods section Digestive enzyme activities and bile acid concentrations). Significant GM effect (*p*<0.05) is indicated over the column and the trends (*p*<0.10) are marked with #. Significant SBM effect and GM-SBM interaction (*p*<0.05) are indicated by differing upper and lower case letters, respectively.

**Figure 2:**
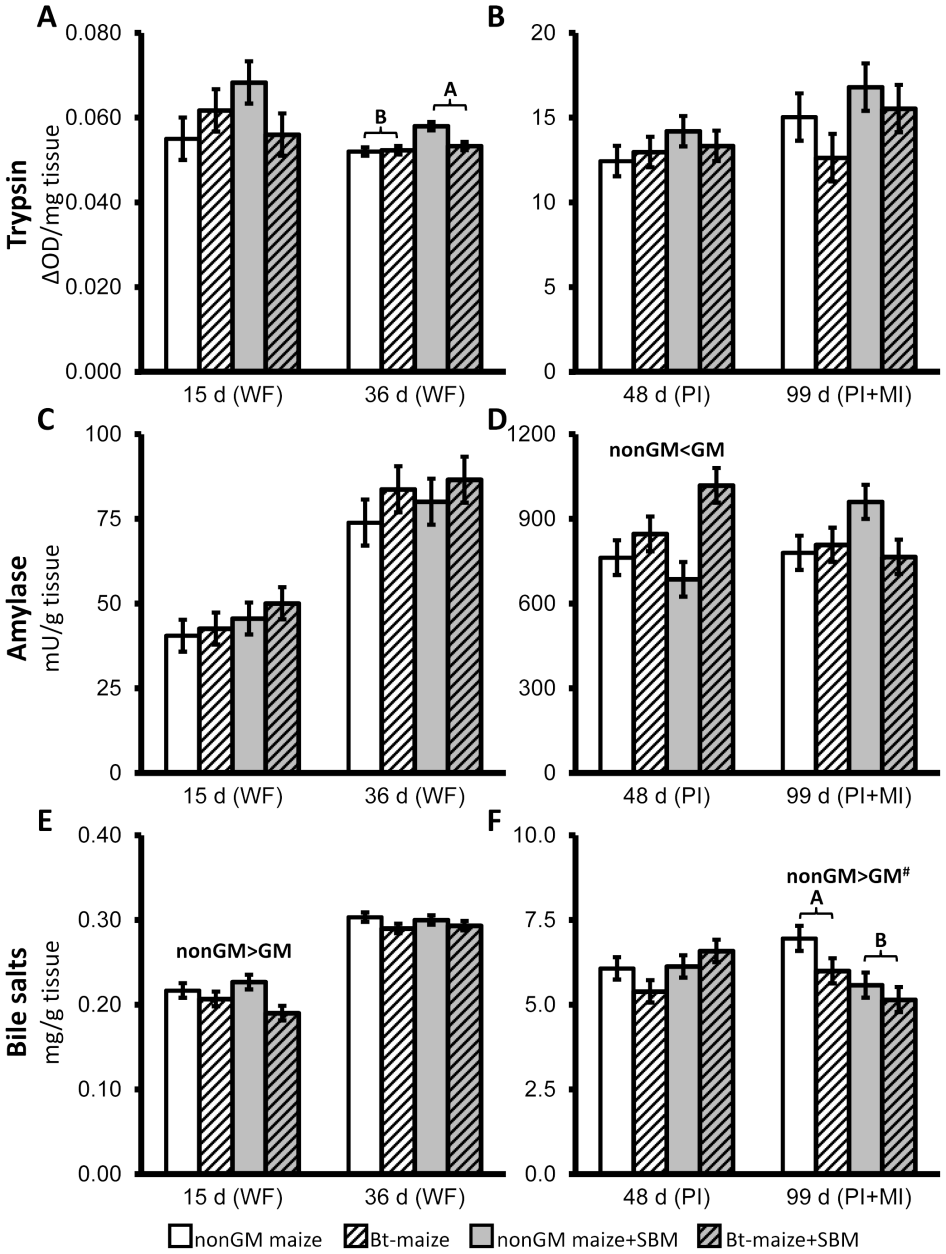
Mean activities of trypsin (A and B) and amylase (C and D) as well as bile salt concentration (E and F) in whole fish (WF) and intestinal sections of Atlantic salmon juveniles fed non-GM maize or GM maize (*Bt*-maize) without or with soybean meal (SBM) from the first-feeding to day 99. Means ± pooled standard errors were calculated from pooled samples of 10–15 fish per tank, three replicate tanks per treatment group (n = 3; for more details see Materials and methods section Digestive enzyme activities and bile acid concentrations). Significant GM effect (*p*<0.05) is indicated over the columns and the trends (*p*<0.10) are marked with #. Significant SBM effect and GM-SBM interaction (*p*<0.05) are indicated by differing upper and lower case letters, respectively.

**Figure 3:**
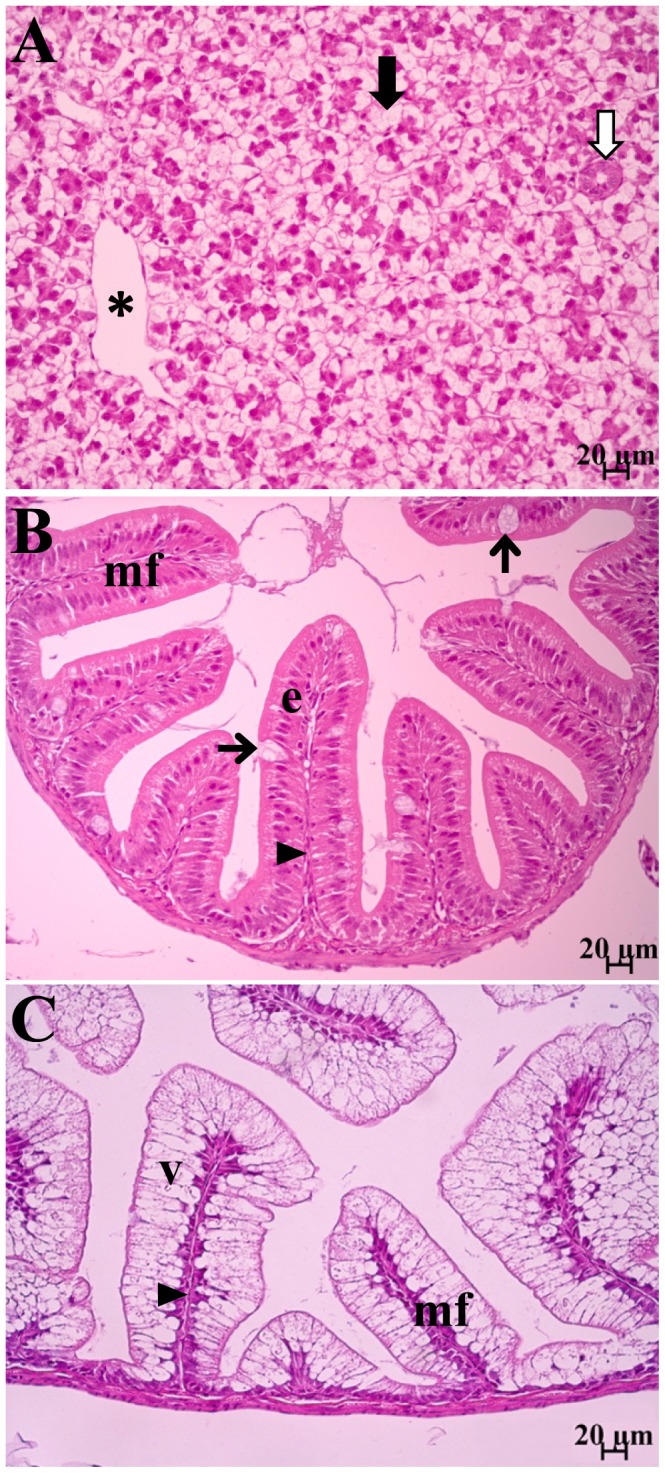
Representative histological detail of liver (A), pyloric caeca (B) and distal intestine (C) in Atlantic salmon juvenile fed GM maize (*Bt*-maize) with soybean meal (SBM) for 99 days. No consistent differences due to dietary treatment were observed. Normal morphology of liver (A) with high level of glycogen deposits (black thick arrow) in hepatocytes; asterisk and white thick arrow illustrating portal vein and bile duct, respectively. Normal morphology of pyloric caeca (B) with distinct mucosal folds (mf), which are comprised of a single layer of normal enterocytes (e) and scattered goblet cells (arrow); the thin lamina propria (triangle) are lined with a single layer of loose connective tissue. Normal morphology of distal intestine (C) with distinct mucosal folds (mf), which are comprised of single layer of highly vacuolated enterocytes (V); enterocyte nuclei are basally located within the cells; the thin lamina propria (triangle) are lined with a single layer of loose connective tissue.

**Figure 4:**
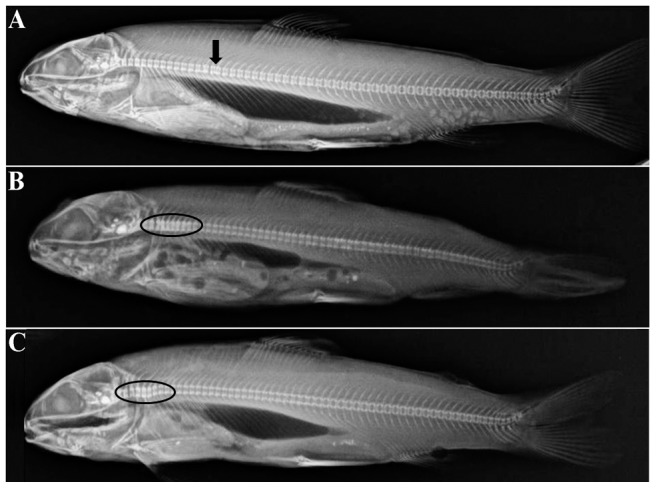
Radiographic examination of the skeleton in Atlantic salmon juveniles fed non-GM maize or GM maize (*Bt*-maize) without or with soybean meal (SBM) for 99 days. The three figures illustrate various deformities observed in five of the 656 fish examined: vertebral fusion (arrow; A) and compressed vertebrae in neck region (oval; B and C). A normal vertebral axis is demonstrated in A and B, while C shows a slight axis deviation. See text for more details.

**Figure 5:**
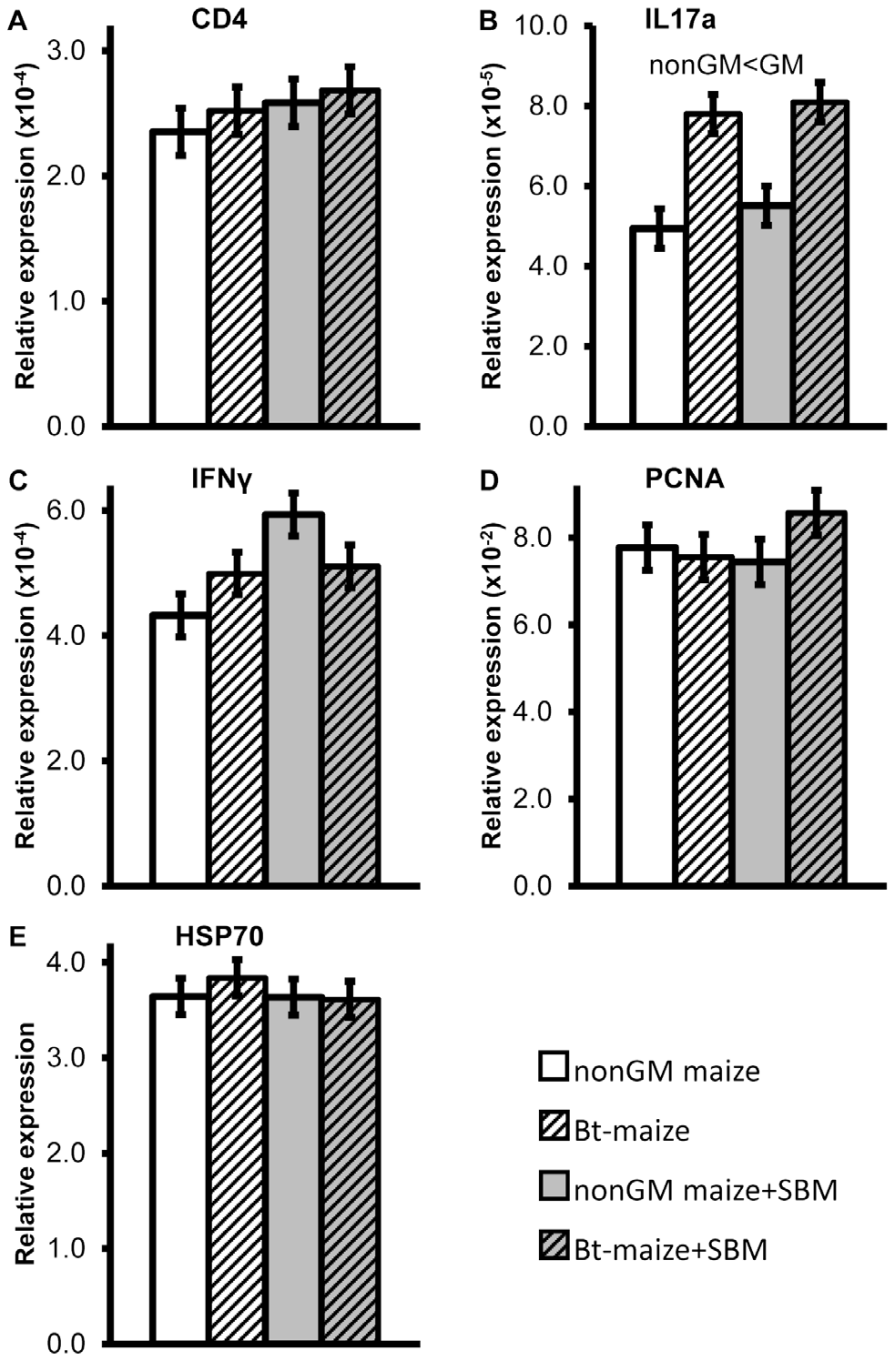
Relative mRNA expression of CD4 (A), interleukin 17a (IL17a) (B), interferon γ (IFNγ) (C), proliferating cell nuclear antigen (PCNA) (D), and heat shock protein 70 (HSP70) (E) in distal intestine of Atlantic salmon juveniles fed the non-GM maize or GM maize (*Bt*-maize) without or with soybean meal (SBM) for 99 days. Means ± pooled standard errors were calculated from the means of three replicate tanks per treatment group (n = 3), with measurements performed on three fish per tank (for more details see Materials and methods section Quantitative real-time PCR [qPCR]). The significant GM effect (*p* = 0.0246) on IL17a expression (B) is indicated over the columns.

During the feeding trial, the SBM-fed Atlantic salmon juvenile generally displayed lower cumulative mortality and enhanced growth performance compared to the other diet groups (Table 3). Total cumulative mortality, as well as mortalities during the periods day 16–36 and day 37–48 were significantly lower in SBM-fed fish. Condition factor was significantly higher on day 15 and 36 and tended (*p*<0.10) to be higher at day 48 and 99 compared with non-SBM fed fish. Moreover, significantly higher body weight, fork length and SGR were observed in SBM-fed fish on day 99. When differences were observed in LAP (Fig. 1A–C) and maltase (Fig. 1D–F) activities in either whole fish or dissected intestinal sections, the SBM-fed groups usually exhibited significantly lower or a trend (*p*<0.10) towards lower activities, with an exception of significantly higher LAP activity in PI on day 48 (*p* = 0.0107; Fig. 1B). Trypsin activity (Fig. 2A–B) in whole fish was significantly higher in fish fed SBM diets on day 36 (*p* = 0.0374; Fig. 2A), whereas amylase activity (Fig. 2C–D) was not affected. Bile salt concentration (Fig. 2E–F) tended to be higher in PI on day 48 (*p* = 0.0955) but was significantly lower in PI+MI on day 99 (*p* = 0.0169) in SBM-fed fish (Fig. 2F). Histological examination revealed that SBM did not induce inflammation in the DI of Atlantic salmon juveniles. The SBM-fed fish displayed normal morphological structures in DI (Fig. 3), with the exception of one of the 24 sampled SBM-fed fish that exhibited mild, focal, inflammatory lesions in the submucosa. No changes were observed in the liver or PI sections, in skeletal morphology (Fig. 4), or in transcriptional expression of CD4, IL17a, IFNγ, PCNA or HSP70 in the DI (Fig. 5).

**Table 3:** Mean cumulative mortality (number of individuals per period and total), growth performance, condition factor and specific growth rate (SGR) of Atlantic salmon juveniles fed non-GM maize or *Bt*-maize without or with soybean meal (SBM) from first-feeding to day 99.

	Normal (non-SBM)		Challenged (SBM)			Two-way ANOVA		
	non-GM maize	*Bt-*maize	non-GM maize	*Bt-*maize		GM	SBM	Interaction
					pSE	*p* value	*p* value	*p* value
**Cumulative mortality**								
Day 1–15	3.3	3.7	5.0	2.3	0.9	0.24	0.86	0.14
Day 16–36	14.7	27.3	11.7	7.7	3.2	0.21	***0.0070***	***0.0296***
Day 37–48	12.7	15.0	9.0	7.0	1.6	0.92	***0.0063***	0.21
Day 49–99	2.3	2.7	0.7	2.0	0.7	0.27	0.14	0.50
Total	33.0	48.7	26.3	19.0	4.3	0.36	***0.0029***	***0.0281***
**Body weight (g)**								
Day 15	0.25	0.26	0.26	0.28	0.02	0.48	0.27	0.92
Day 36	0.64	0.67	0.66	0.65	0.04	0.81	0.94	0.69
Day 48	1.00	1.01	1.05	1.03	0.08	0.88	0.66	0.85
Day 99	3.25	3.14	3.97	4.27	0.22	0.69	***0.0029***	0.38
**Body length (cm)**								
Day 15	2.7	2.7	2.8	2.8	0.04	0.97	0.45	0.91
Day 36	3.6	3.7	3.6	3.5	0.1	0.98	0.45	0.17
Day 48	4.2	4.2	4.2	4.2	0.1	0.91	0.89	0.77
Day 99	6.3	6.2	6.6	6.8	0.1	0.49	***0.0057***	0.43
**Condition factor**								
Day 15	1.19	1.25	1.25	1.29	0.02	***0.0266***	***0.0204***	0.70
Day 36	1.35	1.31	1.38	1.39	0.02	0.65	***0.0452***	0.34
Day 48	1.34	1.32	1.35	1.36	0.01	0.71	***0.0866***	0.39
Day 99	1.26	1.24	1.27	1.29	0.01	0.90	***0.0663***	0.14
**SGR**								
Day 15	2.35	2.75	2.97	3.17	0.38	0.45	0.21	0.80
Day 36	3.69	3.80	3.76	3.70	0.17	0.87	0.92	0.62
Day 48	3.67	3.70	3.80	3.73	0.16	0.90	0.65	0.76
Day 99	2.98	2.94	3.18	3.25	0.06	0.78	***0.0021***	0.38

Means and pooled standard errors (pSE) were calculated from the means of three replicate tanks per treatment group (n = 3), with measurements performed on 10 fish per tank.

Initial body weight (g): 0.17±0.01; initial body length (cm): 2.5±0.1; initial condition factor: 1.04±0.08.

The *p* values are given for the main variables non-GM/GM and non-SBM/SBM inclusion, respectively, as well as *p* values for interactions between the variables by two-way ANOVA analysis.

### Effects of Bt-maize

#### Growth performance

Despite slightly lower crude protein and lipid contents in the *Bt*-maize containing diets (Table 1), differences in mortalities, body weight, fork length, condition factor, and SGR between conventional and *Bt*-maize fed fish were minimal during the trial with the exception of a significantly higher condition factor in *Bt*-maize fed fish on day 15 (Table 3). A significant interaction between GM and SBM effects was observed on total cumulative mortality and mortality during the period day 16–36, indicating lower mortality in fish fed *Bt*-maize with SBM inclusion but not in fish fed *Bt*-maize without SBM (Table 3). No differences in body composition on day 99 were detected (Table 4), indicating similar or slightly improved nutrient utilization and accretion in the diet groups fed *Bt*-maize.

**Table 4:** Mean whole body composition of Atlantic salmon juveniles fed non-GM maize or *Bt*-maize without or with soybean meal (SBM) for 99 days.

	Normal (non-SBM)		Challenged (SBM)			Two-way ANOVA		
	non-GM maize	*Bt-*maize	non-GM maize	*Bt-*maize		GM	SBM	Interaction
					pSE	*p* value	*p* value	*p* value
***Whole body, g 100 g^−1^***								
Crude Protein	16.8	16.5	16.2	16.2	0.4	0.61	0.32	0.68
Crude lipid	6.60	6.57	6.60	6.83	0.11	0.41	0.28	0.28
Ash	2.48	2.48	2.38	2.34	0.08	0.79	0.17	0.86
Dry matter	26.4	26.0	25.4	25.6	0.6	0.85	0.29	0.61
Energy (MJ/kg)*	6.62	6.38	6.39	6.36	0.16	0.41	0.45	0.51

Means and pooled standard errors (pSE) were calculated from pooled samples of 20 fish per tank, three replicate tanks per treatment group (n = 3).

The *p* values are given for the main variables non-GM/GM and non-SBM/SBM inclusion, respectively, as well as *p* values for interactions between the variables by two-way ANOVA analysis.

*Gross energy was calculated using the energy concentrations of 39.5 for lipid, 23.6 for protein, and 17.2 kJ/g for glycogen (glycogen levels were calculated as: 100 – (water + crude protein + crude lipid + ash).

#### Digestive enzyme activities and bile salt concentration

No significant *Bt*-maize effects were observed on LAP activity in either whole fish or intestinal tissues throughout the trial (Fig. 1A–C). However, a significant interaction between GM and SBM effects was observed on day 36 in whole fish, which indicated lower LAP activity in fish fed *Bt*-maize without SBM but not in fish fed *Bt*-maize with SBM inclusion (*p* = 0.0052; Fig. 1A).

*Bt*-maize significantly decreased maltase activity in whole fish on day 36 (*p* = 0.0024) and the same interaction was observed between GM and SBM effects as for LAP activity (*p*<0.0001; Fig. 1D). On day 48, *Bt*-maize fed fish also showed a tendency towards a lower maltase activity in PI (*p* = 0.0502), and in DI+MI an interaction between GM and SBM effects was detected, in which fish fed the *Bt*-maize diet with SBM inclusion displayed significantly lower maltase activity compared to fish fed *Bt*-maize without SBM (*p* = 0.0103; Fig. 1E).

Trypsin activity was not affected by *Bt*-maize either in whole fish or intestinal tract sections during the trial (Fig. 2A–B). Amylase activity in PI was significantly increased due to *Bt*-maize feeding on day 48 (*p* = 0.0095; Fig. 2D). At the earlier sampling points, the same numerical trends were observed. At the final sampling this trend, however, was absent.

Bile salt concentration in fish fed *Bt*-maize was significantly decreased on day 15 in whole fish (*p* = 0.0273; Fig. 2E) and tended to be lower in PI+MI on day 99 (*p* = 0.0982; Fig. 2F).

#### Liver and intestinal histology and skeletal development

Most liver samples exhibited normal morphology with high to medium levels of glycogen deposition (Fig. 3A). In five of the 48 examined samples, slight morphological changes were observed. Three of these displayed mild focal inflammation, and the other two showed mild fatty changes. However, no significant associations with *Bt*-maize were detected.

No morphological changes were observed in PI of any of the examined fish (Fig. 3B). Most DI samples displayed normal morphological structures (Fig. 3C). The exception was slight changes in two of the 48 examined samples. One fish from the *Bt*-maize diet group displayed an increased frequency of goblet cells and unevenly sized absorptive vacuoles. Another fish fed the *Bt*-maize with SBM inclusion diet exhibited mild, focal, submucosal inflammation. However, no significant *Bt*-maize effects were observed.

For skeletal development, five of the 656 examined fish exhibited skeletal deformities. Among those five, one fish in each of the two *Bt*-maize groups exhibited vertebrate fusion (Fig. 4A). The other three fish, one from three different diet groups, displayed neck compression (Fig. 4B and 4C). Thus, no diet effects on skeletal morphology were detected.

#### Expression of selected genes in DI tissue

The relative mRNA expressions of CD4, IL17a, IFNγ, PCNA, and HSP70 in DI on day 99 are shown in Fig. 5A–E, respectively. *Bt*-maize significantly increased IL17a expression level (*p* = 0.0246; Fig. 5B). No interactions between GM and SBM effects were observed.

## Discussion

### SBM effects

The reason for including SBM in the diets in this feeding trial was to investigate whether an intestinal inflammatory response would alter responses to, or alternatively be altered by, simultaneous *Bt*-maize exposure. But the data indicate that SBM-induced inflammation may not be suitable as a challenge model in juvenile salmon. The present study is one of only two reported feeding trials on SBM in diets for Atlantic salmon juveniles from first-feeding [10]–[11], [29]. Even with the higher inclusion level in the current study (16.7 versus 12.5%), SBM did not cause a marked inflammatory response in the DI as has been reported in older post-smolts [30]–[36]. Inclusion levels as low as 7.6% extracted SBM caused detectable, moderate inflammatory changes in post-smolt salmon, while 15.3–27% caused severe changes [33]. Thus the 16.7% inclusion level employed in the current study was expected to cause marked inflammatory changes. The absence of inflammation as assessed histomorphologically was supported by the lack of significant changes in transcriptional expression of immune response parameters such as CD4, IL17a and IFNγ, cellular proliferation (PCNA), and stress (HSP70) markers, which have all been shown to be markedly up-regulated in post-smolt salmon with SBM-induced inflammation [28], [35]–[36]. These results suggest that the Atlantic salmon juveniles at the earliest stages are tolerant of the soybean components that induce DI inflammation in older fish. On the other hand, some mild alterations in digestive function were observed as indicated by reduction in activity of LAP and maltase, as well as bile acid concentration, and increase in trypsin activity at some sampling points. Except for the magnitude, they were generally in line with results of previous studies with SBM in diets for older salmonids [11], [28], [33], [46]. Nevertheless, and in contrast to many studies with older salmonids [28], [33], [47]–[48], survival and growth performance of the SBM-fed juvenile salmon was enhanced, the cause of which is not clear. However, some studies indicate that low levels of saponins can stimulate growth [49]–[52], suggesting that the present positive effect of SBM may be due to its content of saponins. Alternatively, the adaptive immune system in the juvenile fish may still have been under-developed and therefore not functionally equipped to mount an inflammatory response, e.g. the T-cell mediated immune response reported in more developed salmon [28], [35]–[36]. This is supported by vaccination practices in salmon aquaculture, with a recommendation of minimum 15 g body weight at first vaccination to ensure a protective antibody response. Final body weight following the present 99-day feeding trial was ca. 4 g. Further investigations are underway to follow the response to SBM over time as the juvenile salmon's functional immune response develops.

### *Bt*-maize effects

Earlier reports of *Bt*-maize (MON810 event) effects on growth performance in fish has shown varying results. Hemre et al. [25] reported reduction in growth in Atlantic salmon, whereas Sissener et al. [12] and Sanden et al. [14] reported enhanced growth and no effects on survival in zebrafish. The present results, showing no significant GM effects on survival, growth performance or feed utilization (as assessed by body composition analyses), are in line with the results of our earlier *Bt*-maize study in post-smolt salmon, in which the same batch of *Bt*-maize was used [28]. The reduced mortality on day 36 and in total following 99 days of feeding in the groups fed the SBM-containing *Bt*-maize diet further supported absence of any *Bt*-maize or SBM effects on general health in the juveniles.

Although survival and growth did not seem to be affected by the *Bt*-maize, some mild changes in intestinal function were apparent. Similar findings were also observed in our previous study in post-smolts [28]. This may be due to the lectin characteristics of the *Bt* (Cry) protein [53]–[55], which may bind to sugar residues present on the intestinal surface of the fish [5], [15]. Lectin-binding has been reported to interfere with intestinal homeostasis, causing changes in for example intestinal growth, digestive enzyme activities and pancreatic secretion [56]–[57], which may explain our observations regarding the decreased LAP and maltase activities and the generally higher amylase activity. On the other hand, higher amylase activity may also be explained by the slightly higher carbohydrate levels in *Bt*-maize containing diets [58]–[59]. But since nutrient digestibilities of the experimental diets could not be determined in the small fish in this trial, the consequences of the decreased bile salts concentration and digestive enzyme activities on digestion cannot be postulated. However, the absence of *Bt*-maize effects on survival, growth performance and whole body composition suggests that any minor loss of function measured was not severe enough to impair overall fish performance and health and may simply reflect compensatory responses to compositional differences in the two maize meals [37]–[38].

Skeletal deformities can be a problem in intensive salmon farming, especially for first-feeding fish due to their rapid growth, high nutrient requirements and relative lower efficiency of nutrient and mineral utilization [60]. The reduction in mineral digestibility observed in *Bt*-maize fed post-smolt salmon [28] raised concerns regarding the skeletal development in *Bt*-maize fed salmon juveniles. To our knowledge, this was the first study on effects of any GM plant ingredient on animal bone health. The very low frequency of skeletal deformities in general and no differences attributed to *Bt*-maize consumption specifically suggest that *Bt*-maize did not affect bone development of juvenile salmon in this 99 day trial. In addition, the histological examinations indicated that the presence of *Bt*-maize in diets did not induce major impairment to any organs or tissues examined. This conclusion is in line with other studies of effects of GM ingredients in Atlantic salmon [10]–[14], [25]–[29], [34], [48].

Previous mammalian studies have indicated immunogenicity of Cry1A protein [16]–[20]. An *in vitro* digestion trial, in which Cry1Ab was only slightly degraded at pH 2, even at high pepsin-to-substrate ratio [61], has suggested that Cry1Ab protein immunoreactivity may survive passage through the digestive tract. The Cry1Ab protein fragments have been detected in digesta of *Bt*-maize fed pigs [62]–[64]. In Atlantic salmon, the pH range along the gastrointestinal tracts is 4.5–8.6 [65]. Integrity of Cry1Ab protein may thus be assumed to be less modified during the passage through the gastrointestinal tract in Atlantic salmon than in monogastric mammals. In the present study, the increased gene expression of IL17a in DI on day 99 indicated that *Bt*-maize may activate a mild, local IL17a-mediated immune response in juvenile fish. The magnitude of the up-regulation, however, was less than 2-fold and therefore not considered a sign of inflammation in the DI. This was confirmed by the absence of inflammatory changes as assessed by histomorphological evaluation. During SBM-induced inflammation in older salmon, IL17a expression has been reported to increase more than 200-fold [36]. In the previous post-smolt salmon study [28], rather than an IL17a response a transient CD4 response (as observed with Western blot, but not with qPCR) and an increased IFNγ expression following 97 d exposure were observed, which also indicated a mild, possibly transient immune stimulating effect of *Bt*-maize. The different *Bt*-maize effects found in juvenile and post-smolt salmon may be explained by differences in immune responses between the developmental stages, as indicated also by the lack of DI inflammatory response to SBM in the juvenile fish. The differing responses also preclude any conclusions regarding potential biomarkers for *Bt*-maize exposure or responses in Atlantic salmon.

In conclusion, the Atlantic salmon juveniles fed *Bt*-maize for 99 days from first-feeding showed similar survival, growth performance and feed utilization (as assessed by body composition analyses) as those fed diets with the non-GM near-isogenic maternal line. Furthermore, microscopic and radiographic examinations did not reveal negative *Bt*-maize effects on the liver, intestinal tract or skeletal morphology or development. However, the *Bt*-maize diets apparently did somewhat alter digestive function as indicated by significant reductions in LAP and maltase activities and gut bile salt concentrations, and increased amylase activity at some sampling points. IL17a expression in the DI following 99 days of exposure was also significantly increased. But these responses may not be of biological significance since they did not appear to impact general fish health, and may be as much due to compositional differences in the two maize meals [37]–[38] as to the genetic modification and Cry1Ab content. The question of whether *Bt*-maize would change a hypersensitivity response in salmon was not resolved, since the juvenile fish were apparently tolerant to SBM inclusion in the diet.
